# Young age and shorter duration of Crohn’s disease are associated with non-adherence to taking medication

**DOI:** 10.14744/nci.2021.08634

**Published:** 2022-02-10

**Authors:** Hasan Yilmaz

**Affiliations:** Department of Gastroenterology, Kocaeli University Faculty of Medicine, Kocaeli, Turkey

**Keywords:** Age, Crohn’s disease, disease duration, education status, medication adherence

## Abstract

**Objective:**

The mainstay of Crohn’s disease treatment is medical therapy. Failure to comply with medications causes disease activation, loss of response to treatment, and increased hospitalization rates. Drug non-adherence worsens the course of the disease, leading to fistula, stricture, and surgical interventions. The adherence rates to drug therapy in Crohn’s disease patients and the risk factors vary considerably in the literature. The aim of the study was to investigate drug adherence rates and factors affecting adherence to Crohn’s disease medications.

**Methods:**

This study was conducted as prospective cohort study at the tertiary health care institution inflammatory bowel disease outpatient clinic within 1 year. Crohn’s disease characteristics and pharmacy records of consecutive patients were evaluated. Medication adherence was assessed by calculating the medication possession ratio using the amount of medication purchased from the pharmacy.

**Results:**

A total of 129 patients were included in the study. It was observed that 43.6% of the patients did not comply with their Crohn’s disease medications. It was determined that the patients who did not adhere to the medication were significantly younger (41±12 vs. 48±13, p=0.039). The duration of the disease is shorter in patients who did not comply with the drugs (4.50 [IQR: 3.00–12.00] vs. 6.00 [IQR: 3.00–12.00, p=0.025]). Adherence with medication is lower in patients with higher education levels (35.7% vs. 64.3%, p=0.023).

**Conclusion:**

Medication adherence is of critical importance for Crohn’s disease outcomes. Nearly half of Crohn’s disease patients do not comply with drugs. Young and highly educated patients with shorter disease duration should be targeted for measures to increase the rates of medication adherence.

**C**rohn’s disease is a chronic inflammatory destructive bowel disease that can attack the entire digestive tract. It is estimated to affect 1.4 million people in Europe and 1.6 million people in the United States of America [1, 2]. Its incidence was reported 4.4/100,000 in Turkey [3]. Crohn’s disease, characterized by abdominal pain and diarrhea, has life-threatening complications such as intestinal strictures, obstructions, bleeding, intra-abdominal abscess, fistula, and perforation. Up to 47% of patients also have extraintestinal diseases or experience hospitalization and surgery that significantly affect their quality of life (QoL) and long-term outcomes [4]. Roughly half of the patients develop intestinal complications within 10 years after diagnosis and require surgical intervention within 20 years [5]. Factors negatively associated with short- and long-term treatment include but are not limited to access to a gastroenterologist, the traveling distance to hospital, lead time to effective treatment, genetic factors, dysbiosis, side effects of medications, and not adhering to medication. Medical treatment is the mainstay of symptomatic relief, maintenance of remission, and prevention of complications [6].

Not adhering to taking medication was previously found to be associated with flares, relapses, loss of response to medication, decrease in the QoL, morbidity, and mortality. It is also quite common and ranges between 35% and 66% [7, 8]. When inflammatory bowel disease (IBD) patients experienced flare the costs increased 2–3-fold on an outpatient basis. In case of hospitalization due to flares costs increased up to 20-fold [9]. In light of this evidence, it is critical to secure patients’ compliance with medications to enhance disease-related outcomes. Non-adherence to medication is complex and driven by many factors. The factors include young age, the male sex, marital status, employment status, depression, periods of remission, multiple drugs, multiple doses, routes of drug administrations, adverse effects, and the relationships of patients and doctors. These factors were defined in the very heterogeneous cohorts and populations with different cultural heritage. Non-adherence with Crohn’s disease treatments and the success of treatment differs between societies. Thus, outcomes need to be confirmed in the Turkish population and it is necessary to define new factors that could be generalizable.

In this study, it was aimed to determine the rates of non-adherence to taking Crohn’s disease drugs in a tertiary health-care hospital, Turkey. The factors that may affect non-adherence to medication for future targeted interventions for better outcomes of the disease were investigated.

## Materials and Methods

### The Design and Setting of the Study

This study was sub-analysis of research conducted as a single-center prospective cohort study, namely, “Examination of the attitudes of physicians’ who perform online polyclinic examination.” The factors that may affect adherence to medications for Crohn’s disease were analyzed in this study. Patients with Crohn’s disease on active medication at least for a year from June 2020 to June 2021 in the IBD outpatient clinic of a tertiary healthcare facility were included for analysis (the Kocaeli University Medical Faculty Hospital). Patients with inconsistent data in hospital electronic records, patients on Crohn’s disease medication for less than a year, patients under the age of 18 years, and patients with ulcerative colitis or indeterminate colitis were excluded from the study. Those who did not give informed consent, patients with active psychiatric disorders on the sedative and hypnotic drugs, alcohol abusers, and patients with history of sleep disorder were also excluded from the study. Patient demographic information and data that may affect adherence to treatment were the smoking status, disease activity, delay time of diagnosis, level of education, and medications. Disease activity was calculated using the Harvey Bradshaw Index [10]. Scores below 4 were considered as a disease in remission and more than 5 were active diseases. Uncomplicated patients and data on patients with complications such as fistula, stricture, intra-abdominal abscess, and perianal disease were extracted from the hospital electronic record system. The travel distance and duration from the addresses of patients’ residence to the outpatient clinic were calculated using Google Maps. Patients with primary, secondary, and high school education were defined as low educated, and patients with college, university, and doctoral education degrees were defined as highly educated. Lead time for the diagnosis was defined in months between the occurrence of symptoms related to Crohn’s disease and established diagnosis. In other words, it defines the delay time of diagnosis.

**Highlight key points**

• Medication non-adherence in Turkish Crohn’s disease patients is as high as 43.6%.
• Younger patients with a mean age of 41 years significantly did not comply with the Crohn’s disease medications (41±12 vs 48±13, p=0.039).
• The median disease duration is 4.5 years for patients who did not adhere to Crohn’s disease medications and, it is shorter compared to those who adhere to medications with a disease duration of 6 years.
• Those with higher education status are significantly non-adherent to medications.

### Measuring Adherence to Medication

Adherence to taking medication was calculated with the medication possession ratio (MPR). The days and amounts of medication dispensed from pharmacies were retrospectively extracted for the 12 months. The MPR was calculated by dividing the number of drugs purchased from the pharmacy by the number of days during the period of observation. For example, if the patient was prescribed two boxes of 28 tablets on June 1 to use daily and the patient applied for the next prescription 60 days later, the MPR was 56/60=0.93. Patients with an MPR >0.86 were considered adherent to medication. There was no golden standard for adherence to the drug. Methods based on the knowledge of pharmacy refills are quite reliable [11].

### Statistical Analyses

All statistical analyses were performed using IBM SPSS for Windows version 20.0 (SPSS, Chicago, IL, USA). Numeric variables were presented depending on normal distribution with either mean±standard deviation or median and interquartile range (IQR). Categorical variables were summarized as counts (percentages). Comparisons of numeric variables between groups were carried out using independent samples t-test/Mann–Whitney U-test, whichever was appropriate. The association between two categorical variables was examined by the Chi-square test. All statistical analyses were carried out with 5% significance, and a two-sided p<0.05 was considered statistically significant. Logistic regression was performed for multiple independent variable analyses.

### Ethical Considerations

The protocol of this study was reviewed and approved by the Kocaeli University Ethical Committee of Clinical Research (identifier GOKAEK-2021/4.25, project number: 2021/78). This study was conducted following the principles of the Helsinki Declarations revised in 2013.

## Results

### Baseline Characteristics of the Study Population

A total of 155 patients were recruited, but four patients were diagnosed with indeterminate colitis, the home addresses of seven patients were absent, and five patients were excluded due to inconsistent data in the hospital electronic records. As a result, 139 patients were finally included in the analysis. The mean age of the study population was 45±8 years and 71 (52%) patients were women. The patients traveled an average of 89 km for the outpatient clinic examination. Sixty-five (50%) patients arrived the hospital by their vehicles, and patients had visited outpatient clinics 4 times a year on average. Sixty-nine (51.9%) of the study group consisted of highly educated patients. While 5-aminosalicylic acids (5-ASAs) were prescribed to 129 (92.8%) Crohn’s disease patients, it was observed that infliximab (IFX) 16 (11.7%) was used infrequently. Eighty-nine (64%) of the patients in treatment were in remission during the study (Table 1). The overall delay in diagnosis for the study population was 15 months.

**Table 1.** Baseline characteristics of the study population

Variables	
Age*	45.3±13.3
Gender (female) (%)	52.2
Travel distance to outpatient clinic*	89±118
Travel time to outpatient clinic*	78±76
Duration of disease (years)*	6.6±5.6
Lead time to diagnosis	15.3±16.1
Smoking status (%)	
Non-smoker	36.7
Former smoker	27.3
Current smoker	36.0
Outpatient visit last year*	4.2±2.7
Changed residence (%)	7.5
Transport to hospital (%)	
Own car	50.0
Public transport	33.1
Own car and public transport	16.9
Medication (%)	
ASA	92.8
Budesonide	46.3
Azathioprine	61.3
Anti-TNF	25.0
Adalimumab	15.8
Infliximab	11.7
Education (%)	
Low^¥^	48.1
High^§^	51.9
Household income (%)	
Low	39.7
Moderate	86.3
High	13.7
Crohn’s disease behavior (%)	
Complicated	18.7
Not complicated	81.3
Disease activity (%)		
Active	36.49
Remission	64.0

*: Mean±SD; ¥: Education levels lower than high school degree; §: University and college degree; ASA: American Society of Anesthesiologist; SD: Standard deviation.

### Adherence to Crohn’s Disease Medications

Overall 43.6% of our study population were found not to adhere to taking medications. The mean MPR was 0.78 when all treatments were considered. No statistically significant difference was observed between individual drugs in terms of adherence; 5-ASA, azathioprine (AZA), adalimumab (ADA), and IFX (Table 2). In the scope of potential parameters that may be related to adherence, it was observed that younger patients had lower adherence to medication in the univariate analysis (41±12 vs. 48±13, p=0.07). It was also detected that patients had low adherence to treatment than those who had the disease shorter duration (4.50 [IQR: 3.00–12.00] vs. 6.00 [IQR: 3.00–12.00], p=0.025). Regarding the effect of education on adherence to treatment, it was observed that patients with higher education had significantly lower adherence (35.7% vs. 64.3%, p=0.023) (Table 2).

**Table 2.** Univariate analysis of the factors associated with medication non-adherence

	Adherent	Non-adherent	p
Age*	48±13	41±12	0.039
Gender (%)			1.00
Female	52.0	51.7	
Male	48.0	48.3	
Travel distance to outpatient clinic (km)**	49	10	
Travel time to outpatient clinic (km)**			
Duration of disease (years)**	4.5	6	0.025
Smoking status (%)			0.412
Non-smoker	42.7	31. 0	
Former smoker	26.7	31.0	
Current smoker	30.7	37.9	
Outpatient visit last year (%)			
Changed residency	94.6	89.7	0.334
Transport to hospital (%)			1.00
Own car	50.7	49.1	
Public transport	32.9	33.3	
Both	16.4	17.5	
Medication (%)			
ASA	92.0	93.1	1.00
Budesonide	47.3	45.6	0.86
Azathioprine	68.9	54.4	0.128
Anti-TNF (%)			
Adalimumab	18.7	13.8	0.607
Infliximab	9.3	16.1	0.371
Education (%)			0.023
Low^¥^	56.0	35.7	
High^§^	44.0	64.3	
Household Income (%)			0.972
Low	40.5	38.6	
Moderate	45.9	47.4	
High	13.5	14.0	
Crohn’s disease behavior (%)			0.609
Complicated	17.3	22.4	
Not complicated	82.7	77.6	
Disease activity (%)			0.999
Active	34.7	36.2	
Remission	65.3	63.8	

*: Mean±SD; **: Median; km: Kilometer; ¥: Education levels lower than high school degree; §: University and college degree; SD: Standard deviation; ASA: American Society of Anesthesiologist; SD: Standard deviation.

There was no significant association between the parameters of distance from the hospital, means of transportation to the hospital, smoking status, individual drugs, having active or complicated disease, household income, and adherence to taking medications. An independent factor that could predict adherence could not be determined by a logistic regression analysis (Table 3).

**Table 3.** Multivariable logistic regression analysis of risk factors for adherence to medications in patients with Crohn’s’s disease

	SE	Wald	p	OR	95% CI	
					Lower	Upper
Age	0.016	2.185	0.139	1.024	0.992	1.057
Duration of disease	0.038	2.300	0.129	1.060	0.983	1.143
Education	0.401	1.920	0.166	0.574	0.262	1.259
Constant	0.814	1.097	0.295	0.426

SE: Standard error; OR: Odds ratio; CI: Confidence interval.

## Discussion

One of the most important and maybe the first step to the success of treating Crohn’s disease is patients’ adherence to the drug therapy. Adherence to drug therapy was traditionally defined as the usage of more than 80% of the medications prescribed to the patient in the recommended doses and intervals. In this study, the rates of non-adherence to taking medication and the factors that may affect adherence were identified in Turkish Crohn’s disease patients. To the best of our knowledge, this was the first study focusing on rates of non-adherence to taking medications and related factors in a Turkish Crohn’s disease patients cohort.

Non-adherence rates to taking Crohn’s disease medications were found 42.3% in our cohort. Non-adherence to AZA was the highest at 68.9% and the lowest to IFX at 9.3%. The study result was revealed younger age and higher education associated with lower adherence, while the long-lasting disease was associated with higher adherence to taking Crohn’s disease medications.

Other studies report non-adherence rates between 30% and 43% based on questionnaires, patients’ reports, or visual analog scales. This rate ranges between 17.5 and 45% when the MPR is calculated according to the number of pharmacy refills. In our study regarding individual drugs, the adherence to AZA was at 68.9%, to ADA at 18.7%, and to IFX at 9.3%. Previously, the rate of non-adherence was reported at 35–72% to AZA [12], 43% to ADA, and 7% to IFX [13]. The rates of non-adherence in our study were comparable to the literature, and it can be concluded that the results of this study could be generalizable for Turkish Crohn’s’s disease patients. In concordance with the previous reports, it was also found young age was associated with non-adherence [14]. This phenomenon could be explained with the reasoning that young patients mostly from the working class and busy at work can easily forget to take medications. In addition, being young and restless could drive one to careless behavior or individuals could be reckless and insouciant. One other reason can be that young patients may underestimate their conditions and ignore taking medications as they do not yet experience complications related to the disease. Clinicians should thus pay extra attention to young Crohn’s disease patients and motivate them to take medications.

The results of our study are in agreement with the majority of the previous studies – longer duration of disease was associated with adherence to taking medication [15–18]. It is presented as an independent indicator of non-adherence in case of short duration of the disease according to D’inca et al. [17] Another explanation is that patients who have not been exposed to the disease in the earlier stages may not attribute importance to the disease and consequently opt out of taking medications. The symptoms in early stages of the disease are mostly milder and this could also lead patients to negligence. In the long-term patients who were frequently exposed to flares and complications may stick to the drugs not to experience them again. In a recent study, new users of anti-TNF were found less adherent to treatment [18]. This could be because awareness of the disease could develop in time. On the contrary, a study revealed that adherence to oral IBD medication is associated with shorter duration of disease [14]. While oral therapy is frequently used by clinicians in patients with lower disease activity or remission, parenteral drugs are more often preferred in severe diseases. Patients in treatment for a long time may experience a higher rate of remission with the effect of treatment, and they may think that they do not need the drugs anymore because they feel better and because of the milder symptoms, they can stop oral treatment. Patients should be warned that there may not always be a correlation between symptoms and disease activity in Crohn’s disease and therefore not to become complacent and discontinue treatment.

High education was associated with non-adherence [19]. Moreover, Coenen et al. [20] found higher education as an independent predictor of non-adherence to taking medications. In agreement with the previous research, higher education was also associated with lower adherence to taking Crohn’s disease medications in this study. There can be several reasons for this outcome. First, it can arise from higher education being associated with lower social control. Highly educated people can be more independent in society and easily escape the surveillance of their families and social circles. This may cause them not to be accountable to anyone when they do not comply with treatment and therefore not non-complying with treatment. Second, highly educated people, along with high self-esteem, can generate arguments and develop resistance to recommended treatment. In patients with lower education in most countries, doctors are the most knowledgeable people and the experts of the disease, thus people with low education unquestioningly obey the treatment culturally. It should be ensured that all patients, especially those with high education, receive sufficient information about the disease, drug treatment, side effects of medications, and alternative treatments. They should be treated as stakeholders in the treatment instead of the paternalistic approach. Good patient-physician relations and successful communication have been shown to increase adherence to taking medications [21].

Although the previous studies noted significant associations of adherence to medications with gender, smoking, household income, and disease behavior, this study did not find any relation between adherence and those parameters. It was also hypothesized that barriers to reaching effective health care could have an impact on adherence, but neither travel distance nor means of transportation were statistically significant. Further large multicenter studies are needed to discover the relation.

This study has some limitations. The patient population consists of a relatively small number of subjects, which brings the risk of not detecting small statistical differences. Multicentered larger studies could generate generalizable results for the Turkish population. The MPR was used to measure adherence instead of a questionnaire or direct detection of drug metabolites or serum levels. The MPR is based on the pharmacy refill count and more reliable than a questionnaire for the detection of adherence. However, MPR results are limited to detecting whether non-adherence is intentional where there are questionnaire-based powers to detect psychosocial preferences. Finally and unfortunately, we currently do not have the opportunity to use direct measuring methods.

### Conclusion

The study results demonstrated non-adherence to taking medications was high in patients with Crohn’s disease. Young patients with long duration of the disease and high education have high non-adherence to taking Crohn’s disease medications. In Crohn’s disease, incompliance with treatment may result in serious complications, physicians should focus on following up with patients in the risk groups of not adhering to taking medications.

### Acknowledgements:

The author is grateful for assisting in data extraction to Dr. Burcu Kocyigit (Department of Internal Medicine, Kocaeli University School of Medicine) and valuable support in statistical analysis to Dr. Sibel Balci (Department of Biostatistics, Kocaeli University School of Medicine).
